# Fully automatic HER2 tissue segmentation for interpretable HER2 scoring

**DOI:** 10.1016/j.jpi.2025.100435

**Published:** 2025-03-18

**Authors:** Mathias Öttl, Jana Steenpass, Frauke Wilm, Jingna Qiu, Matthias Rübner, Corinna Lang-Schwarz, Cecilia Taverna, Francesca Tava, Arndt Hartmann, Hanna Huebner, Matthias W. Beckmann, Peter A. Fasching, Andreas Maier, Ramona Erber, Katharina Breininger

**Affiliations:** a Pattern Recognition Lab, Department of Computer Science, Friedrich-Alexander-Universität Erlangen-Nürnberg, Erlangen, Germany; b Institute of Pathology, University Hospital Erlangen, Erlangen, Germany; c Department of Gynecology and Obstetrics, Universitätsklinikum Erlangen, Friedrich-Alexander-Universität Erlangen-Nürnberg, Erlangen, Germany; d Department Artificial Intelligence in Biomedical Engineering, Friedrich-Alexander-Universität Erlangen-Nürnberg, Erlangen, Germany; e Center for AI and Data Science (CAIDAS), Universität Würzburg, Würzburg, Germany; f Institute of Pathology, Klinikum Bayreuth GmbH, Bayreuth, Germany; g Surgical Pathology Unit, Azienda Sanitaria Locale, Presidio Ospedaliero, Ospedale San Giacomo, Novi Ligure, Italy; h Institute of Pathology, University Regensburg, Regensburg**,** Germany

**Keywords:** HER2, Histopathology, Deep learning, Semantic segmentation, HER2 scoring

## Abstract

Breast cancer is the most common cancer in women, with HER2 (human epidermal growth factor receptor 2) overexpression playing a critical role in regulating cell growth and division. HER2 status, assessed according to established scoring guidelines, offers important information for treatment selection. However, the complexity of the task leads to variability in human rater assessments. In this work, we propose a fully automated, interpretable HER2 scoring pipeline based on pixel-level semantic segmentations, designed to align with clinical guidelines. Using polygon annotations, our method balances annotation effort with the ability to capture fine-grained details and larger structures, such as non-invasive tumor tissue.

To enhance HER2 segmentation, we propose the use of a Wasserstein Dice loss to model class relationships, ensuring robust segmentation and HER2 scoring performance. Additionally, based on observations of pathologists' behavior in clinical practice, we propose a calibration step to the scoring rules, which positively impacts the accuracy and consistency of automated HER2 scoring. Our approach achieves an F1 score of 0.832 on HER2 scoring, demonstrating its effectiveness. This work establishes a potent segmentation pipeline that can be further leveraged to analyze HER2 expression in breast cancer tissue.

## Introduction

Breast cancer represents the most common malignant disease diagnosed in women, with approximately 2.25 million new cases and 685,000 related deaths reported worldwide in 2020.^1^ The disease encompasses multiple subtypes driven by distinct molecular mechanisms, each offering tailored therapeutic strategies.^2^ Among these, human epidermal growth factor receptor 2 (HER2)-positive breast cancer, characterized by overexpression of the HER2 receptor, accounts for approximately 15–20% of cases and is associated with more aggressive tumor behavior.^3^

Targeted therapies against the HER2 receptor are well-established and have been shown to improve patient survival.^4^ Clinical decisions regarding anti-HER2 treatments typically rely on a patient-specific HER2 expression score. Pathologists determine this score by examining immunohistochemically stained tumor tissue sections and applying the American Society of Clinical Oncology (ASCO)/College of American Pathologists (CAP) guidelines, which assign each tumor cell to one of four HER2 categories (0, 1+, 2+, or 3+) based on membrane staining patterns.^5^ Aggregating these cell-level categories yields an overall HER2 score that guides therapy selection: a score of 0 is considered HER2 negative, 1+ is HER2-low, 3+ is HER2 positive, and 2+ is equivocal and requires further testing to determine whether it should be classified as HER2 positive or HER2-low.^6^ Although this manual process is standard practice, it places a substantial burden on pathologists. It is time-consuming, prone to inter-rater variability,^7^ and complicated by heterogeneity in HER2 expression within the tumor.^8^ Accurate and reproducible scoring is further challenged by the need to distinguish invasive from non-invasive tumor regions, as non-invasive carcinoma should be excluded from the scoring process.

Deep learning methods have recently gained traction in histopathology image analysis, consistently surpassing traditional approaches.^9^ However, existing HER2 scoring approaches each have limitations that hinder their adoption. Membrane segmentation techniques^10^, ^11^, ^12^ provide detailed visualization of membrane staining but demand extensive, detailed annotations and subsequent post-processing to derive cell-wise HER2 categories. These methods typically do not differentiate between invasive and non-invasive regions, preventing end-to-end scoring in full-slide images (whole slide images (WSIs)). In contrast, HER2 cell detection and classification methods^13^, ^14^, ^15^, ^16^ reduce annotation requirements and circumvent the need for post-processing, yet still require single-cell annotations and fail to exclude non-invasive tumor components.

Weakly supervised and patch-based methods^17^, ^18^, ^19^, ^20^, ^21^ considerably lower annotation effort but sacrifice fine-grained detail. As a result, they limit subsequent analyses at the cellular level, which is valuable for investigating intra-tumoral heterogeneity. Some of these approaches demand preselected regions of interest (ROIs) containing only invasive tumor tissue,^17^^,^^19^ undermining fully automated solutions. Others rely on patient-level labels,^18^ making it difficult to confirm that the model adheres to the required exclusion of non-invasive tissue without manual oversight.

To address these shortcomings, we propose a fully automated HER2 scoring system for WSIs. Our approach relies on pixel-level semantic segmentation with polygon-based annotations, requiring a moderate annotation effort while preserving both fine-grained details and larger tissue structures. Crucially, our method incorporates the exclusion of non-invasive carcinoma to conform to ASCO/CAP guidelines, thereby eliminating the need for preselected ROIs. The resulting segmentation enables not only automated HER2 scoring but can also aid in the identification and analysis of intra-tumoral heterogeneity, potentially enabling future insights that extend beyond binary treatment decisions. Finally, we compare our segmentation-based scores with pathologist-assigned labels to better understand the sources of discrepancy, leading to calibrated scoring rules, and more robust clinical assessments.

## Material and methods

### Data

**Fig. 1:**
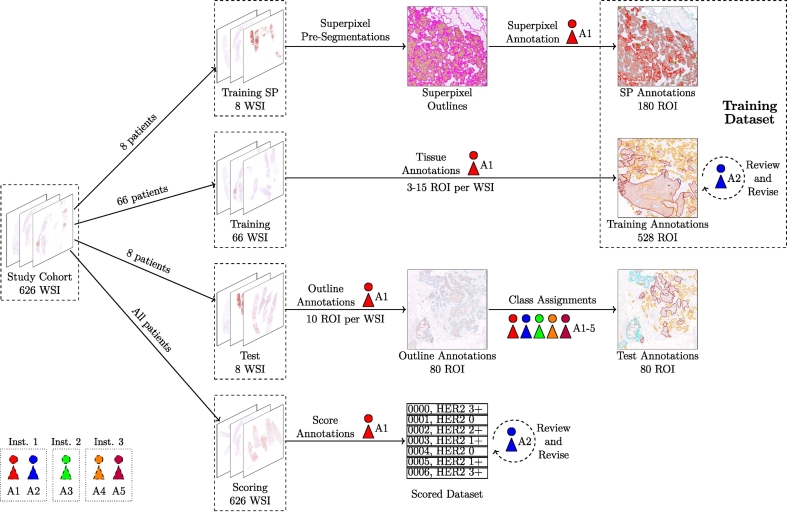
Overview of the annotation procedure for all data used in this work. In total, data from 626 patients is utilized in this work.

An overview of the data and annotation steps employed in this study is presented in Fig. 1. In total, 650 tissue samples were collected from 650 individual patients undergoing routine clinical punch biopsies of breast lesions at the same hospital. All samples were obtained before the initiation of any treatment. Of these, 24 samples were excluded due to the absence of invasive tumor tissue in the WSIs, resulting in a final study cohort of 626 samples.

All patients in this dataset either gave informed consent as part of the Bavarian Breast Cancer Cases and Controls study (#2700), as part of the Imaging and Molecular Detection of Breast Cancer study (#325_19 B) or the retrospective use of FFPE tumor samples (#297_17_Bc).

All samples were prepared using standard tissue preparation and the same immunohistochemical staining protocol (4B5 monoclonal antibody (Ventana, Roche)) to assess HER2 expression, then digitized using the same 3DHistech PANNORAMIC 1000 scanner with a 20× objective lens (0.5 μm/pixel). The resulting WSIs were uploaded to the EXACT annotation platform^22^ for scoring and annotation.

Five annotators contributed to the annotation process. This team included a medical student (A1) with histopathology experience who received training from pathologist (A2) at Institution 1. Additionally, one pathologist (A3) from Institution 2 and two pathologists (A4, A5) from Institution 3 participated, ensuring a diverse set of annotators with high levels of expertise and various institutional backgrounds. Annotators A2–A5 are all board-certified and have profound expertise in breast pathology (A2 > 14 years, A3 > 15 years, A4 > 10 years, A5 > 15 years).

#### HER2 score annotations

**Fig. 2:**
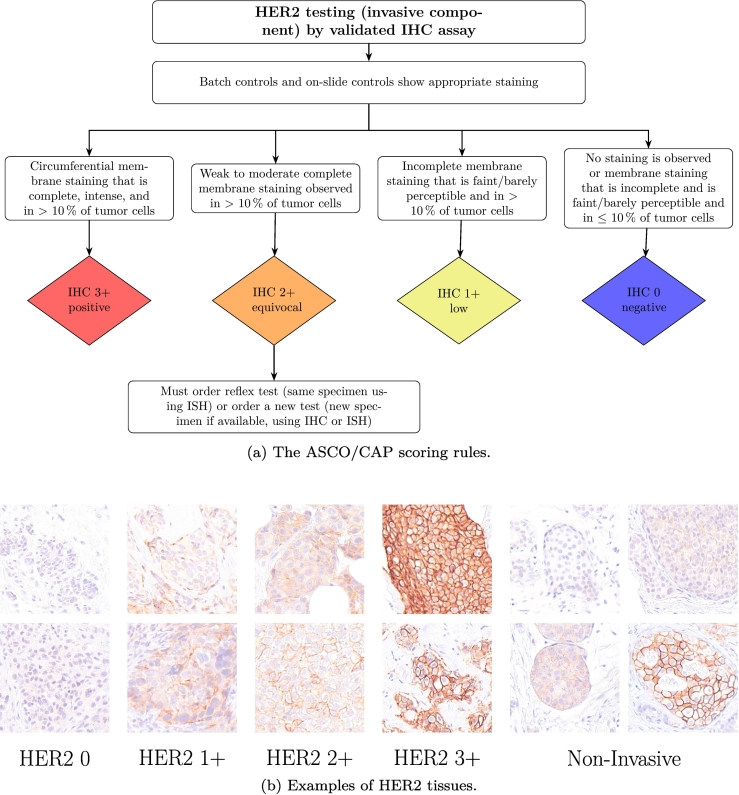
(a) The ASCO/CAP scoring guidelines^5^^,^^6^ for determining patient-wise HER2 scores based on invasive tumor cells. (b) Example tissue images illustrating all four invasive HER2 expression levels (HER2 0: no staining or faint/incomplete staining, HER2 1+: incomplete, faint staining, HER2 2+: weak to moderate complete staining, HER2 3+: complete, intense circumferential staining) as well as non-invasive tumor tissue, which can express any level of membrane staining.

Fig. 2a outlines the ASCO/CAP guidelines used in clinical practice to determine HER2 expression. According to these guidelines, all invasive tumor cells are evaluated for HER2 membrane staining intensity and completeness, and each cell is assigned to one of four categories (0, 1+, 2+, or 3+). Non-invasive tumor cells must be excluded, as their inclusion could erroneously alter the overall HER2 score. Examples of tissues representing these categories are shown in Fig. 2b. Although non-invasive tumor tissue may display HER2 staining, it must not influence the final patient score. The final HER2 score is derived from the distribution of all invasive tumor cells across these categories, with scores of 0 considered HER2 negative, 1+ considered HER2-low, 3+ considered HER2 positive, and 2+ regarded as equivocal, requiring additional tests.^6^

The initial scoring was performed by A1 and subsequently reviewed and revised by A2. Of these 626 WSIs from the study cohort, 98 were categorized as HER2 0, 233 as HER2 1+, 194 as HER2 2+, and 101 as HER2 3+. From the 626 WSIs, 8 WSIs were picked for superpixel (SP)-based training annotation and 74 WSIs (66 for training and 8 for testing) were selected for detailed regional tissue annotations to support segmentation model training, calibration, and further analysis of adjusted scoring rules. The primary selection criterion for these samples was the HER2 score. Equal distribution of HER2 scores was maintained for the SP-annotation slides and the segmentation test set. For the segmentation training set, the proportion of HER2 2+ cases was intentionally increased, as this category was anticipated to be the most critical for model performance.

The remaining 544 WSIs were used to test the scoring algorithms developed in this work. This subset consists of 79 HER2 0 cases, 216 HER2 1+ cases, 161 HER2 2+ cases, and 88 HER2 3+ cases. From the 161 HER2 2+ cases, 106 were diagnosed as HER2 positive after FISH testing was performed.

#### HER2 tissue training annotations

Tissue annotations aimed to achieve both accuracy and efficiency. Instead of labeling each cell individually, polygon-based annotations were employed, which were later converted into pixel-wise segmentation masks suitable for supervised learning. Annotators were instructed to delineate contiguous regions of tumor tissue displaying a uniform HER2 class, leveraging the tendency for adjacent cells to share similar expression characteristics. In cases of mixed expression, more granular polygon annotations—down to the level of single cells—were used as needed.

Given the large amount of tissue in WSIs, annotating entire slides by hand would have been both time-consuming and of diminishing returns. To optimize effort, we utilized ROI based annotation as well as SP based annotation.

*ROI-based annotation.* Pathologist A2 selected 528 ROIs, each measuring approximately 1 mm^2^, that captured a representative diversity of tissue structures and HER2 expression patterns. The medical student (A1) performed the initial polygon annotations, and A2 subsequently reviewed and refined these annotations where necessary.

In total, 42,308 polygons were generated across the training WSIs. Of these, 36,632 encompassed invasive tumor regions—9855 labeled as HER2 0, 16,695 as HER2 1+, 6901 as HER2 2+, and 3181 as HER2 3+ and 902 polygons corresponded to non-invasive tumor tissue (156 lobular carcinoma in situ and 746 ductal carcinoma in situ). The remaining 4774 polygons were assigned to various non-tumor backgrounds, aggregated into a single background class.

*Superpixel-based annotation.* To include some fully annotated patient data, we employed a SP-based annotation approach for eight patients. First, we pre-segmented the entire WSIs using a HER2-specific SP algorithm proposed in,^23^ which generated outlined SPs corresponding to distinct tissue regions. Annotator A1 then assigned class labels to these SPs, refining or adjusting their boundaries as needed. Although this method was less time-consuming than manual annotation from scratch, it introduced a slight reduction in annotation accuracy due to the SP algorithm's initial segmentation quality.

From these SP-based annotations, we sampled a total of 180 ROIs, selecting 30 ROIs for each of the 6 classes to ensure a well-distributed and balanced training set. This approach provided an efficient yet diverse set of annotations.

The full training set for the segmentation algorithm consists of 528 polygon-annotated ROIs and 180 SP-annotated ROIs, resulting in a total of 708 annotated ROIs.

#### Multi-annotator HER2 tissue test annotations

Medical image annotations often exhibit significant inter-rater variability.^24^^,^^25^ To better evaluate the developed algorithms, a multi-annotator test set was created. Five annotators (A1–A5) from three institutions and with various experience levels participated, providing polygon-based annotations similar to those used for training.

To limit their workload while still capturing tissue variability, 10 ROIs were selected per patient by A2, prioritizing diverse tissue types and HER2 patterns. Still, annotation remained time-intensive for experienced pathologists. Moreover, precise boundary delineation is subject to individual interpretation and effort. Given that the main goal was to assess variations in HER2 class assignments rather than boundary detail, a two-step approach was adopted. First, A1 created polygon outlines and deliberately over-segmented ambiguous regions. These polygons were then provided to the other annotators with a default classification. The remaining annotators were encouraged to adjust labels or modify polygons if necessary, ensuring that the primary focus remained on the consistency of HER2 class assignments rather than outline precision.

#### Finding consensus and rater agreements

To determine consensus among the five annotators, all polygon annotations were converted into pixel-wise segmentation masks. Direct majority voting at the class level is not optimal, because the majority could misrepresent the tissue type, especially because one annotator (A1) has less experience than the other four annotators, and could introduce some bias. For instance, a scenario where three annotators each propose a different HER2 class, whereas two annotators label the region as non-invasive tissue could yield a misleading majority vote. Thus, a hierarchical strategy was used. First, consensus was established among three categories: background, non-invasive tumor tissue, and an aggregated invasive tumor class. If the region was invasive, the final HER2 category was then decided by the median of the assigned HER2 grades.

**Fig. 3:**
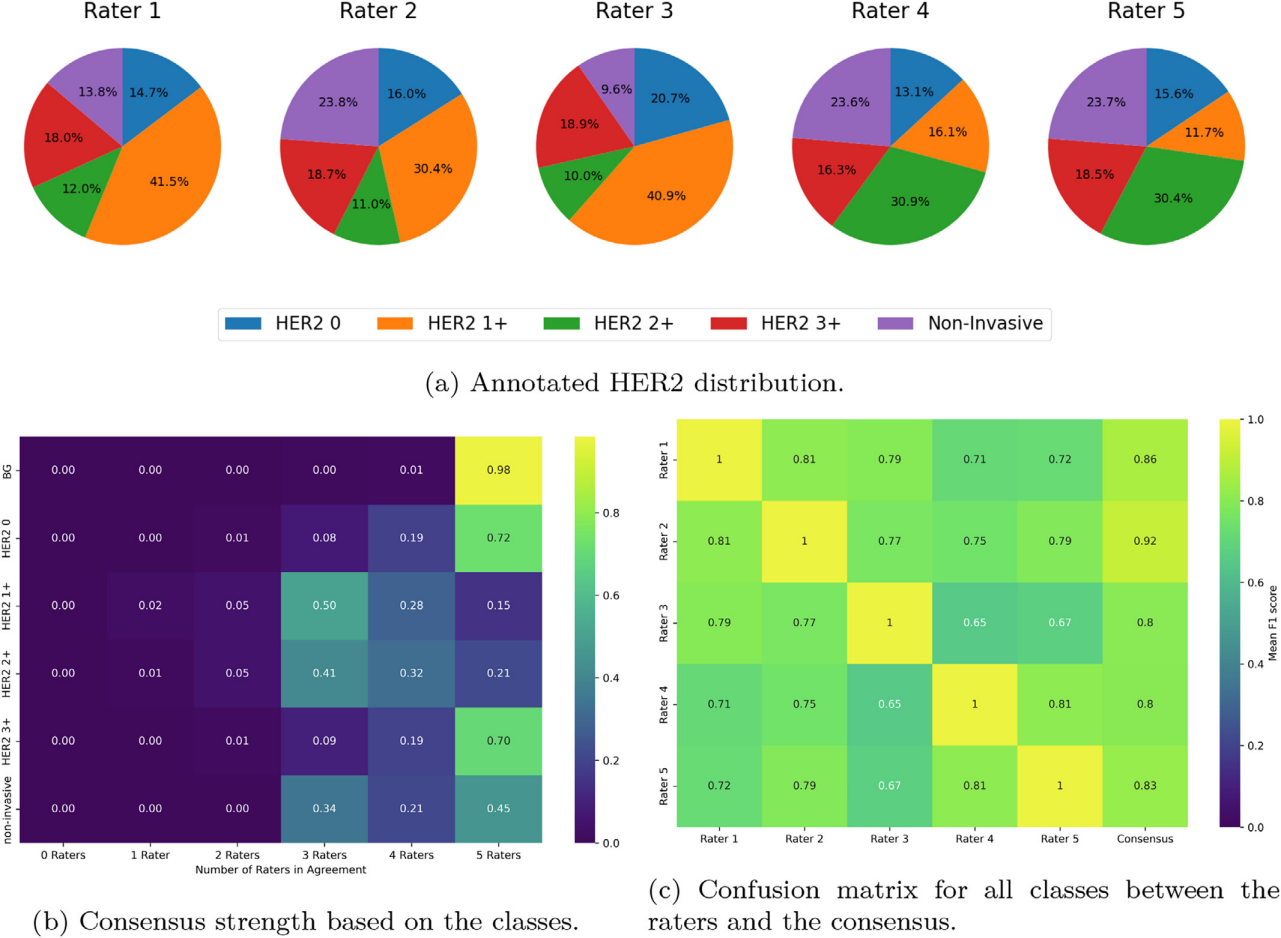
(a) The distribution of the four HER2 classes and non-invasive tumor tissue as annotated by the five raters, illustrating the variability in labeling across different annotators. (b) Visualization of the agreement with the consensus for all classes. (c) A confusion matrix displaying the mean F1 score (average of class-wise F1 scores), comparing each rater to every other rater and to the consensus, thereby highlighting the level of agreement and disagreement among raters.

Fig. 3a compares the distribution of annotated classes across raters, revealing substantial discrepancies for non-invasive tumor tissue as well as for the borderline categories HER2 1+ and HER2 2+. These discrepancies are further illustrated in Fig. 3b, which shows the level of agreement among raters based on the annotated classes. For HER2 1+, HER2 2+, and non-invasive tumor tissue, the raters frequently disagree, resulting in a weak consensus for these categories. This variability likely stems from challenges in reliably identifying non-invasive lesions and the subjective interpretation of borderline staining intensities.

**Fig. 4:**
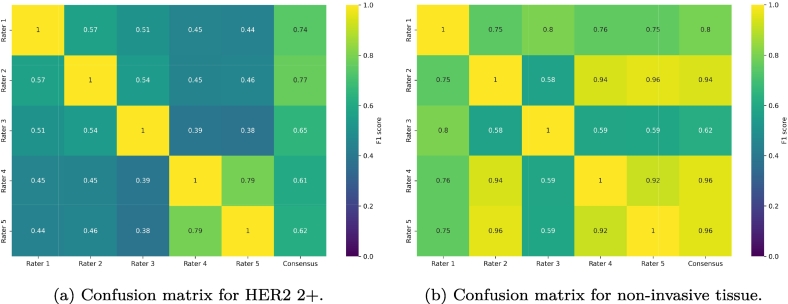
Confusion matrices of the F1 scores between raters and their consensus for HER2 2+ tissue (a) and non-invasive tumor tissue (b).

Fig. 3c shows the mean F1 scores for tumor class annotations among all raters and between each rater and the consensus. As expected, substantial disagreement is evident. This is particularly pronounced when examining the F1 scores between raters for HER2 2+ (Fig. 4a) and non-invasive tumor tissue (Fig. 4b).

Generally, annotators from the same institution exhibit higher agreement with one another, suggesting that institutional standards or familiarity influence annotation consistency. When comparing each annotator to the consensus, higher F1 scores are typically observed, indicating that the consensus protocol effectively mitigated individual biases and produced more robust and reliable annotations.

### Segmentation pipeline

Our segmentation pipeline builds on nnU-Net,^26^ a widely used baseline in medical image segmentation,^27^, ^28^, ^29^ including applications in histopathology.^30^ Key advantages of nnU-Net include its well-tuned training parameters, comprehensive data augmentation strategies, and a sliding window inference approach with Gaussian-weighted overlap. Nevertheless, certain challenges remain when adapting it to our HER2 segmentation task.

#### Histopathology adjustments

Whereas nnU-Net is effective in many medical imaging domains, certain settings in histopathology require modifications.^30^ One significant aspect is the choice of normalization. nnU-Net's use of instance normalization (IN) works well for domains like MRI or X-ray, where image content is relatively consistent. In histopathology, however, the variability in tissue content and scale within each patch can be substantial. If large portions of a patch contain sparse information (e.g., mostly background with a few cells), IN may inflate feature activations and lead to unstable training or label switching at inference time.

To address this, we follow recommendations in^30^ and employ batch normalization (BN) instead of IN. As long as the batch size remains sufficiently large, BN provides more stable normalization across diverse patch contents.

Unlike,^30^ we do not apply hematoxylin–eosin–diaminobenzidine augmentations. Altering staining components can distort the crucial intensity and completeness of membrane staining, which directly determines the HER2 class. Instead, we rely on nnU-Net's default color and contrast augmentations, which maintain a more realistic representation of membrane staining patterns.

In addition, we refined the border padding strategy. nnU-Net's default zero-padding creates dark borders that are uncharacteristic in histopathology slides, which typically have bright backgrounds. We replace this with reflection padding, ensuring that the padded regions closely resemble actual tissue content and thus maintain spatial integrity.

#### HER2 adjustments

A key challenge in HER2 segmentation is the considerable rater variability and label noise, particularly for neighboring HER2 classes (e.g., 1+ vs. 2+) and in identifying non-invasive tumor tissue. The ambiguous boundaries between classes mean that the network is compelled to memorize noisy annotations rather than learning robust class-defining features.

To mitigate the impact of label noise, we propose a modified loss function that incorporates class relationships. Whereas nnU-Net's standard combination of cross-entropy and Dice loss treats all confusions equally, we replace the Dice loss with a Wasserstein Dice loss.^31^ This loss enables us to assign different penalties depending on the severity of misclassifications. For example, misclassifying a HER2 1+ cell as 2+ is considered a less severe error than confusing a HER2 3+ cell with background. Similarly, mixing non-invasive and invasive tumor tissue is penalized less than misclassifying either as background. This approach allows us to embed domain knowledge into the training objective, stabilizing the learning process despite inevitable annotation inconsistencies.

#### Post-processing

Another challenge arises from identifying large non-invasive tumor regions. Differentiating non-invasive from invasive tumor tissue often requires examining the growth pattern of entire cell clusters, rather than individual cells. Patch-based inference methods, such as those used by nnU-Net, have a limited field-of-view, making it impossible for the network to leverage broader structural context.

Whereas multi-resolution networks^32^^,^^33^ could integrate coarse global context, these architectures significantly increase computational costs. Given our large dataset, we opted for a more computationally efficient post-processing strategy. We rely on the fact that non-invasive tumor tissue typically appears as a connected region distinct from other tissue categories.

First, patches containing the boundary of non-invasive tissue are usually segmented correctly due to available contextual cues. Given a suitable patch size and overlapping inference windows, most non-invasive regions will have at least some correctly segmented boundary patches. After segmentation, we perform connected component analysis to identify distinct tissue regions. If a region contains a sufficiently large proportion of non-invasive tumor pixels, we label the entire connected component as non-invasive tissue. This approach leverages structural continuity while avoiding the complexity of multi-resolution architectures.

### Scoring procedure

We derive HER2 scores directly from the final WSIs segmentation masks. Our scoring rules mirror the ASCO/CAP guidelines (Fig. 2a), with the assumption that pixel proportions approximate cell proportions. Excluding non-invasive regions, the final HER2 score is determined by examining the relative area of invasive tumor pixels in each HER2 class:

• **HER2 3+**: >10% of invasive tumor pixels have a HER2 3+ label.
• **HER2 2+**: >10% of invasive tumor pixels are HER2 2+ or higher.
• **HER2 1+**: >10% of invasive tumor pixels are HER2 1+ or higher.
• **HER2 0**: ≤10% of invasive tumor pixels are HER2 1+ or higher.

In practice, pathologists may adopt a more conservative approach when classifying borderline cases (e.g., preferring to assign a HER2 2+ score to ensure follow-up tests). To account for this potential bias, we calibrate the HER2 2+ threshold to 5% in our experiments. Nonetheless, if the underlying staining patterns lie at the borderline between HER2 1+ and 2+, adjusting thresholds alone may not fully capture this clinical behavior.

### Experiment setup

We adopted nnU-Net^26^ as the basis for our segmentation pipeline, leveraging its well-optimized training parameters, extensive data augmentation techniques, and sliding-window inference with Gaussian-weighted overlaps. Based on nnU-Net's adaptive configuration, we employed a patch size of 512 × 512 and down-scaled images to a 5× resolution (down-sampled by a factor of 4, 2.0 μm/pixel) to balance the visibility of fine details with sufficient spatial context.

To enhance the segmentation performance, we used the ResEnc M encoder,^34^ which has been shown to outperform the original encoder without substantial increases in computational load. All models were trained using a 5-fold cross-validation scheme. For final predictions, we combined results from all five folds (ensemble) and applied sliding-window inference without test-time augmentations to reduce computational demands.

The Wasserstein Dice loss was configured to reflect class relationships. Penalties for confusion between HER2 classes increased with their distance along the HER2 scale: 0.25 for neighboring classes, 0.50 for a two-class difference, and 1.00 for a three-class difference. Misclassifying non-invasive tumor as invasive (and vice versa) incurred a penalty of 0.50, whereas confusion with the background was penalized at 1.00.

During post-processing, tissue components with at least one-third non-invasive tumor segmentation were designated as non-invasive. For whole-slide inference, we employed foreground detection to skip empty or near-empty image patches, using a fifth percentile intensity threshold of 250 for white-background uint8 images.

## Results and discussion

### HER2 segmentation performance

We evaluated segmentation quality using three metrics: (1) *Mean F1 score*, averaged over all five tissue classes (HER2 0, 1+, 2+, 3+, and non-invasive tumor), (2) *Cohen's Kappa*, comparing predicted and consensus HER2 classes with linear weighting, and (3) *Concordance F*1, which treats a prediction as correct if it matches any of the five annotators' labels, rather than only the consensus.

Table 1 summarizes the segmentation results. Each incremental modification to the baseline nnU-Net—incorporating histopathology-specific adjustments, HER2-specific adjustments, and final post-processing—improved performance, raising both mean F1 score and Concordance F1, and generally enhancing Cohen's Kappa. Notably, post-processing had minimal impact on Cohen's Kappa, indicating that improvements in segmenting non-invasive tumor tissue had less influence on the final HER2 distribution.

**Table 1:** Results for our segmentation pipeline. The left side of the table shows the results when the five folds are ensembled, whereas the right side shows the average and standard deviation for the individual fold.

	Ensemble			5-folds		
Method	Mean F1 score	Cohen Kappa	Concordance F1	Mean F1 score	Cohen Kappa	Concordance F1
nnUNet	62.98	78.51	83.73	62.10 (1.87)	77.38 (2.68)	82.10 (1.67)
+ Histo. adjustments	65.41	79.83	85.41	62.70 (3.15)	78.95 (0.84)	81.46 (3.54)
+ HER2 adjustments	66.39	80.58	86.19	64.50 (1.04)	79.51 (0.70)	83.35 (1.52)
+ Post-processing	67.73	80.58	87.35	65.81 (0.96)	79.59 (0.74)	84.54 (1.36)

The Concordance F1 was consistently significantly higher than the mean F1 score. This suggests that when the model's predictions differed from the consensus, they often aligned with at least one annotator's opinion. In other words, the network's predictions typically represented a plausible interpretation of the data, rather than a complete misclassification.

Table 2 presents the results of different penalty settings for the Wasserstein Dice loss. Overall, the results demonstrated robustness when adjusting penalties for HER2 confusions. Stronger penalties led to an improvement in Cohen's Kappa, whereas weaker penalties slightly reduced it. However, both adjustments resulted in a decline in the mean F1 score. Additionally, increasing penalties for confusions between non-invasive and invasive tissue proved to be disadvantageous.

**Table 2:** Investigation of the impact of various penalty weights in the Wasserstein Dice loss. We examine three configurations: (1) *weaker regression penalties*, which reduce penalties for HER2 confusions by half (0.125 for 1-off, 0.25 for 2-off, and 0.5 for 3-off errors); (2) *stronger regression penalties*, which double the penalties relative to the default weighting (0.5 for 1-off, 1.0 for 2-off, and 2.0 for 3-off errors); and (3) *stronger non-invasive penalties*, which impose full penalties (1.0) for confusions between invasive and non-invasive tissues. The results highlight the sensitivity of the model to these weight adjustments and their implications for segmentation accuracy.

Method	Mean F1 score	Cohen Kappa	Concordance F1
Default	67.73	80.58	87.35
Weaker regression penalties	66.59	80.39	87.00
Stronger regression penalties	66.89	81.22	86.67
Stronger non-invasive penalties	65.75	79.15	86.05

**Fig. 5:**
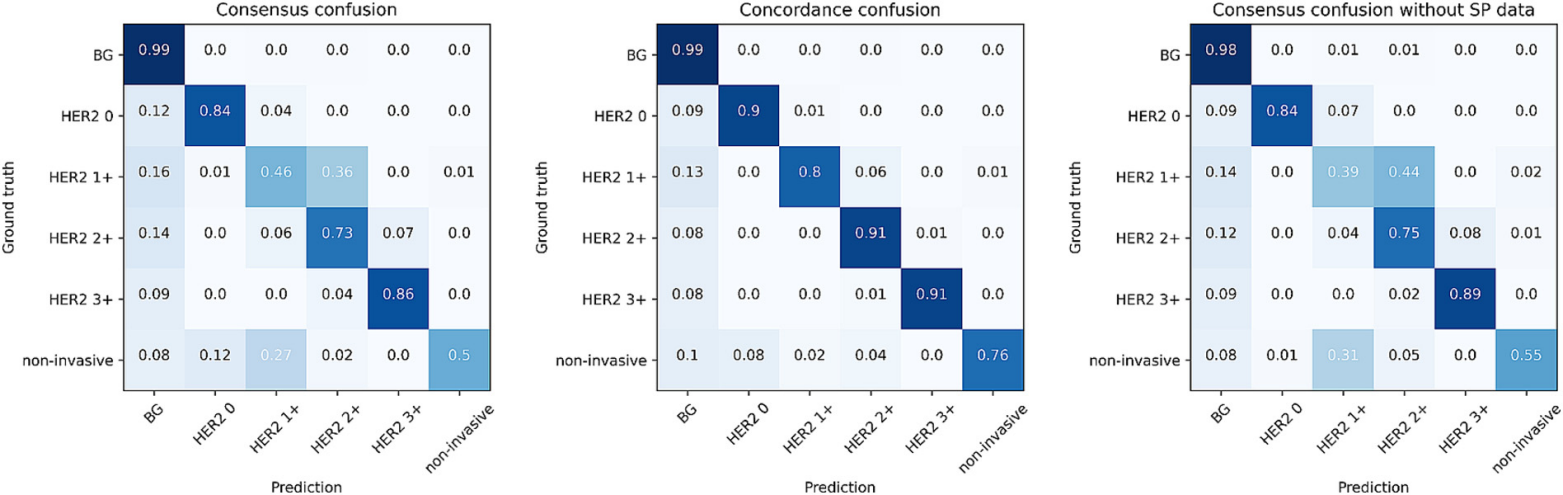
Confusion matrices of our final segmentation method. Left shows the confusion between the prediction between the consensus ground truth. Middle shows the concordance confusion, where a prediction is considered correct if one annotator agreed with the label. Right shows the confusion matrix if our final method is trained without the SP annotated data.

Fig. 5 shows normalized confusion matrices comparing the final model's predictions to the consensus (left) and to any annotator's opinion (middle). The largest discrepancies involved the over-assignment of HER2 2+ where the consensus labeled HER2 1+, and the difficulty in correctly identifying non-invasive tumor tissue (often misclassified as HER2 1+). However, these disagreements largely disappeared when considering concordance with any annotator, reflecting the inherent uncertainty among pathologists. On the right side of Fig. 5, we also present the confusion matrix for our method trained without the SP-annotated data. This configuration results in slightly reduced performance, primarily due to lower segmentation accuracy for HER2 1+ tissue. The mean F1 score decreases to 65.69 (−2.04), and Cohen's Kappa decreases to 79.95 (−0.63), highlighting the benefits of incorporating SP-annotated data into the training process.

**Fig. 6:**
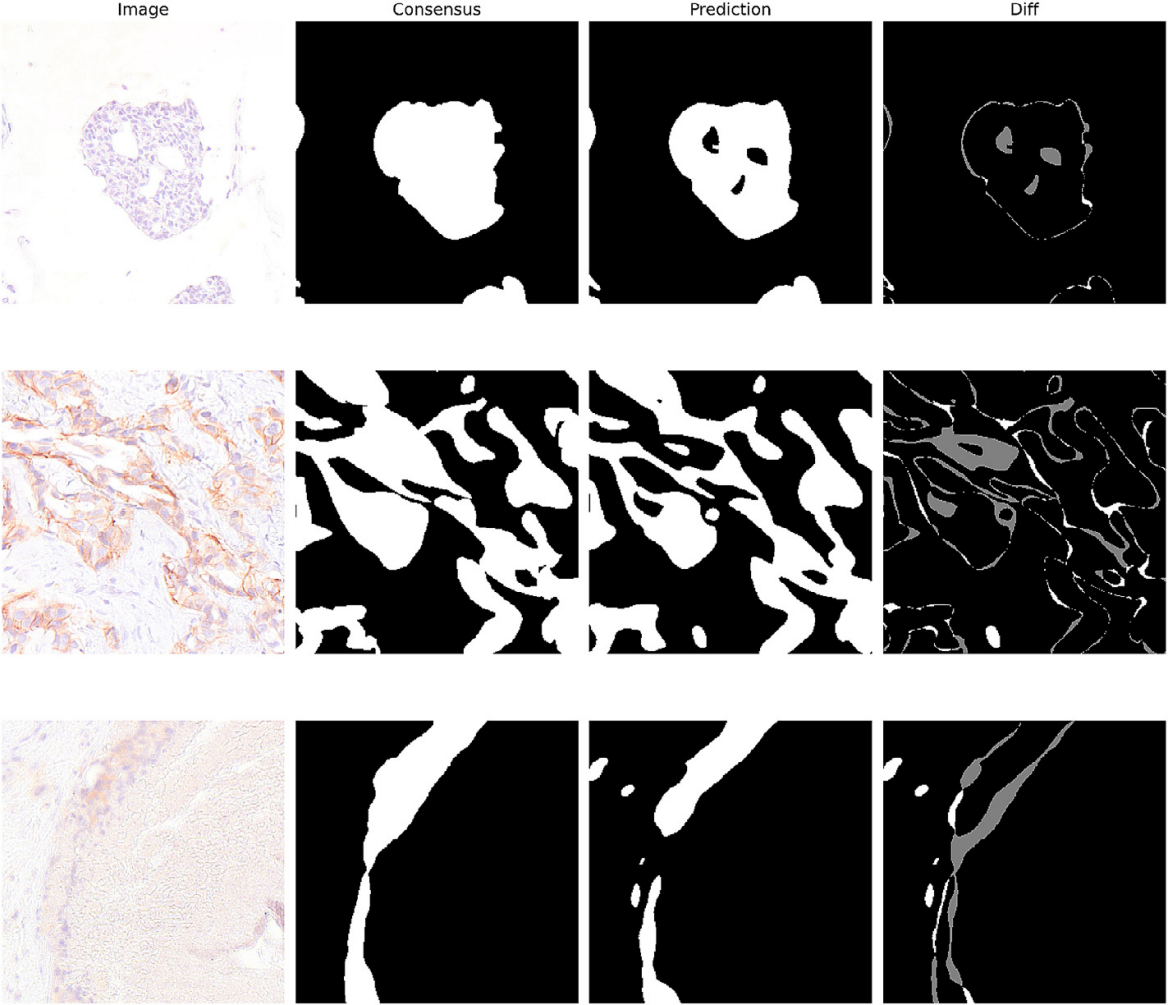
Examples of discrepancies between the annotator consensus and the model prediction with regard to the segmentation of non-background tissues.

The most significant remaining discrepancy for our final method is the misclassification of non-background pixels as background by the model. As illustrated in Fig. 6, this issue is primarily caused by the coarseness of the annotations, such as when small background areas within larger tumor regions are not annotated but are correctly segmented by the model.

**Fig. 7:**
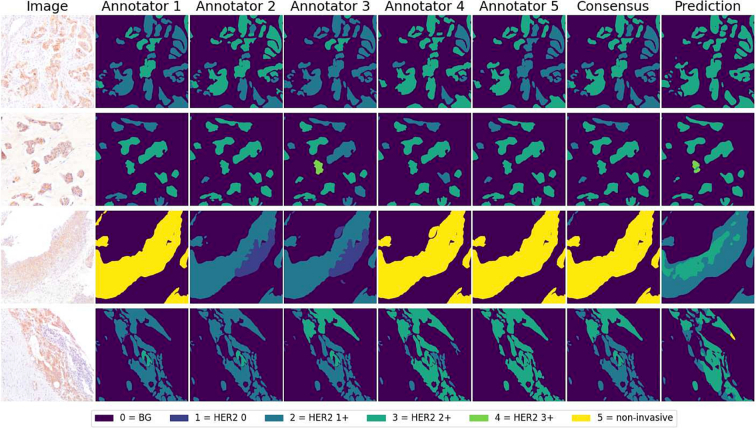
Visual examples of four cases where a disagreement between the annotators is visible, as well as the consensus and the network predictions.

Fig. 7 provides examples illustrating how the model's predictions sometimes deviate from the consensus while still matching certain annotators' opinions. Although Annotator 1 provided the training data, the model's predictions did not show a systematic bias toward this annotator, indicating that the observed variations are more likely due to genuine inter- and intra-annotator variability.

### HER2 WSI scoring performance

Table 3 presents the WSI scoring results obtained from the segmentation methods evaluated in this study. The minor performance differences among these methods indicate that the scoring task is robust, with only a few cases showing sensitivity to segmentation quality. Overall, our final method demonstrates the highest performance, although the improvement due to the post-processing step is observed in only a single instance.

**Table 3:** WSI scoring results for the different segmentation methods.

Method	Mean F1 score	Cohen Kappa	Correct predictions	Accuracy
nnUNet	79.05	77.59	419	77.02
+ Histo. adjustments	79.97	78.47	424	77.94
+ HER2 adjustments	80.69	79.19	428	78.68
+ Post-processing	80.86	79.38	429	78.86

**Fig. 8:**
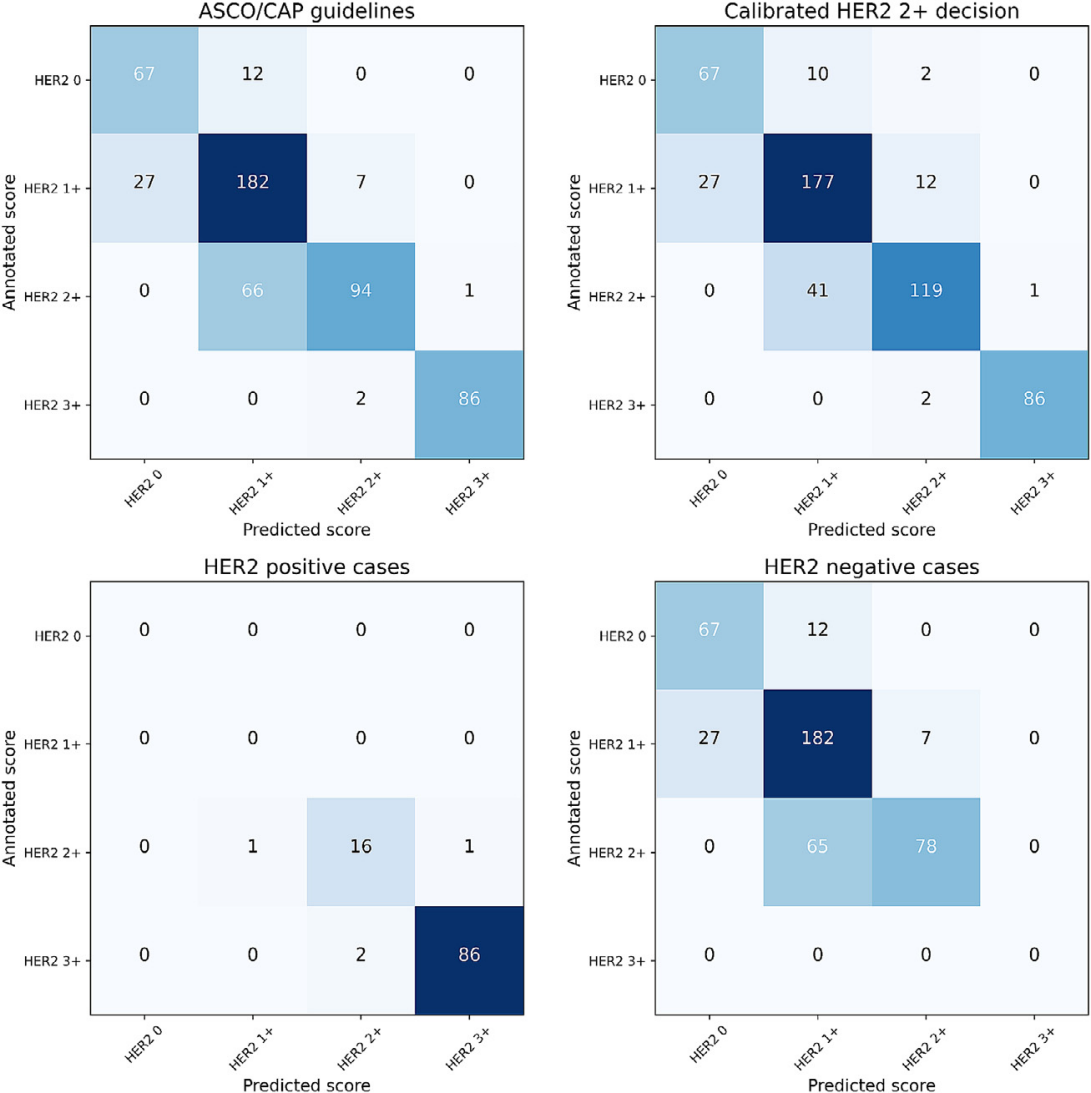
Confusion matrices for our scoring pipeline, when directly following the ASCO/CAP guidelines on all the WSI (upper left) and when only considering HER2 positive cases (lower left) and HER2 negative cases (lower right). Result with the adjusted HER2 2+ thresholds are shown in the upper right.

Fig. 8 presents results for whole-slide scoring according to the ASCO/CAP guidelines based on the results of the full segmentation pipeline. Under these conditions, 429 out of 544 cases (78.86% true-positive rate) were correctly classified, with a mean F1 score of 80.89. The main cause of confusion was the misclassification of HER2 2+ cases as HER2 1+.

When evaluating the scoring results with respect to HER2 status, the model demonstrated high sensitivity and specificity. Among 106 FISH-confirmed HER2-positive cases, 105 were correctly predicted as HER2 2+ or higher. Similarly, all FISH-confirmed HER2-negative cases were accurately predicted as HER2 2+ or lower. The primary source of confusion occurred between annotated HER2 2+ scores and predicted HER2 1+ scores. However, because these cases were ultimately classified as HER2-negative based on FISH testing, the model did not err in terms of HER2 status predictions. Instead, it effectively reduced the number of cases requiring FISH testing: only 106 predicted equivocal cases required FISH testing, compared to 166 annotated cases. Notably, there was one false-negative prediction, where a HER2-positive case was incorrectly predicted as HER2-negative.

Next, we evaluated the model's performance in scoring HER2-low cases (annotated as HER2 1+ and HER2 2+ with negative FISH test). Out of the 359 HER2-low cases present in the dataset, 247 were directly scored as HER2 1+ by the model, whereas 85 would require FISH testing for determination. This resulted in an accuracy of 92.45% for classifying HER2-low cases.

Finally, we adjusted the HER2 2+ threshold from 10% to 5%, reflecting a potential clinical tendency to lean on the side of labeling borderline cases as HER2 2+. With this modification, 449 out of 544 cases (82.54% true-positive rate) were predicted in agreement with the annotated scores, and the mean F1 score rose to 83.97. This change primarily improved the recognition of HER2 2+ tissue.

Table 4 shows results when applying a safety margin to the scoring thresholds. Cases whose predicted HER2 ratios did not exceed the threshold by this margin were excluded due to uncertainty. As the safety margin increased, fewer cases remained, but both the true-positive rate and mean F1 score improved. For example, at a 5% margin, 12% of the cases were excluded, but the true-positive rate reached 84.55% and the mean F1 score improved to 85.88, indicating that uncertain cases often reduced the overall accuracy.

**Table 4:** HER2 scoring metrics, when a safety margin is added to the scoring thresholds, only taken cases in consideration where the predicted ratios exceeded the scoring threshold by the safety margin.

Safety margin	0%	1%	2%	3%	4%	5%	6%	7%	8%	9%
Included cases	544	534	519	506	493	479	465	451	433	404
Inclusion ratio	1.00	0.98	0.94	0.93	0.91	0.88	0.85	0.83	0.80	0.74
Correct predictions	449	442	430	421	411	405	396	385	373	351
Accuracy	82.54	82.77	82.85	83.20	83.37	84.55	85.16	85.37	86.14	86.88
Mean F1 score	83.97	84.17	84.26	84.60	84.68	85.88	86.46	86.69	87.30	88.38

### Interpretability of HER2 scoring

**Fig. 9:**
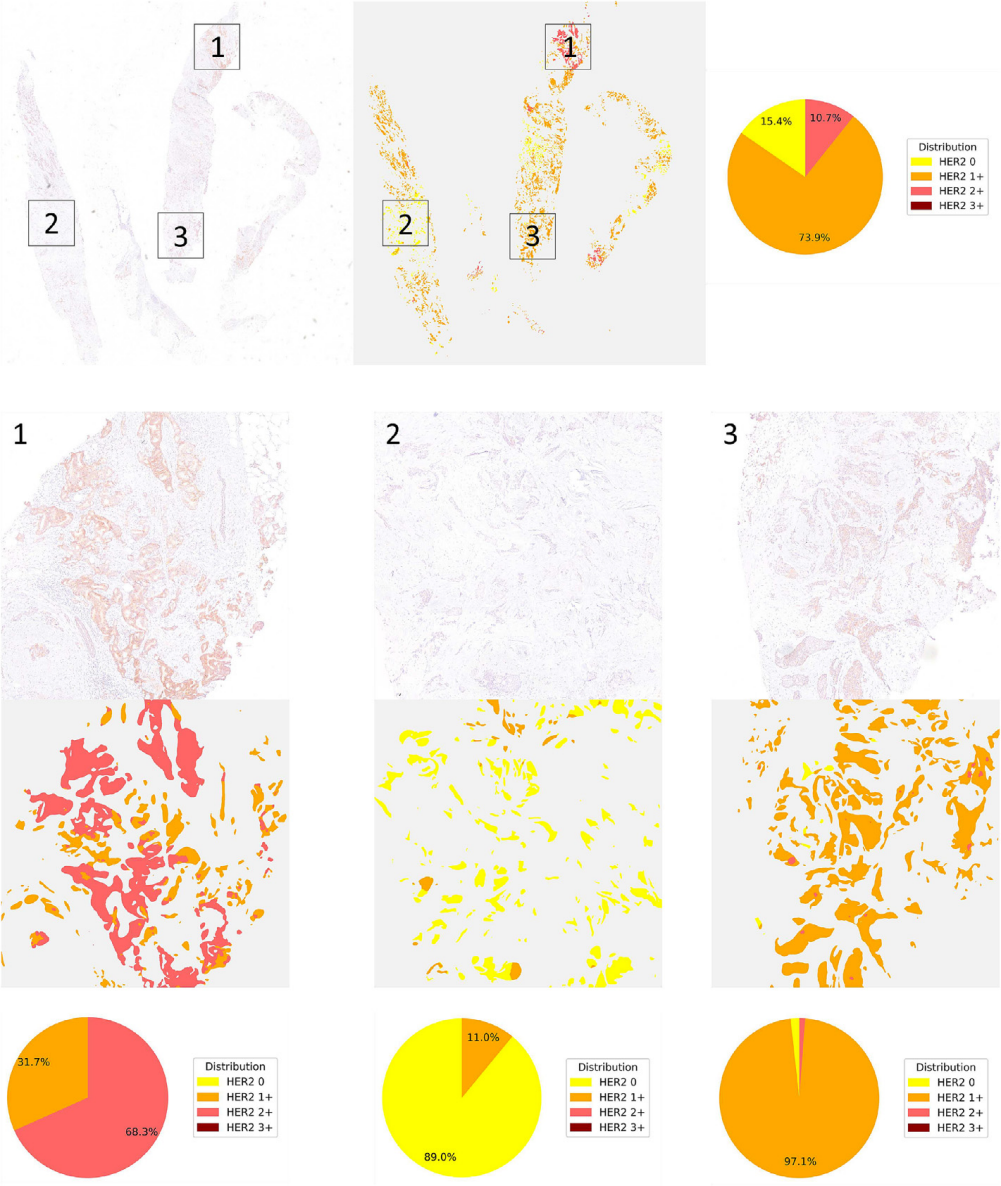
Example illustrating how segmentation results can enhance the interpretability of HER2 scoring. The figure shows the segmentation of a single WSI, along with the overall HER2 distribution derived from it. Three distinct regions within the WSI are highlighted, each exhibiting a different HER2 expression pattern. The corresponding HER2 distributions for these regions are also displayed, demonstrating how local variations in staining intensity and completeness can be easily visualized and analyzed.

Fig. 9 demonstrates how segmentation-based HER2 scoring can enhance interpretability, particularly in cases with heterogeneous HER2 expression. In the presented example, a single patient's tissue displays multiple HER2 staining intensities in different regions. By visualizing these variations in a spatially explicit manner, pathologists can more easily identify and understand why a certain score was assigned. Such insights are challenging to gain from an aggregated score alone, where heterogeneous patterns might be overlooked or misinterpreted.

This spatially fine-grained information not only clarifies how the final HER2 score is derived, but also offers opportunities for interactive refinement. For instance, a pathologist may notice that certain tissue regions are out-of-focus and should be excluded from scoring based on clinical guidelines, or may identify areas that are misclassified as non-invasive or incorrectly assigned a particular HER2 class. With a segmentation-based approach, the pathologist can adjust the identified regions and, if desired, recalculate the HER2 score. In this way, the pipeline can serve as a decision support tool rather than a standalone solution, allowing experts to incorporate their domain knowledge and resolve discrepancies in borderline cases.

### Discussion

A central challenge in this study was the substantial label noise present in the HER2 data, a problem that became increasingly clear when we constructed the multi-annotator test set. Despite this complexity, our segmentation model proved sufficiently robust even when trained on annotations initially generated by one annotator with limited experience and subsequently refined by an experienced annotator.

Although the model's performance was relatively low when measured against the consensus, it notably aligned with at least one annotator's opinion in cases where it did not match the consensus. One reason for the gap in performance is that annotators benefited from predefined polygon-based outlines, guaranteeing perfect spatial alignment among raters. By contrast, our model generated segmentations from scratch, introducing additional spatial variability. Interestingly, the model did not consistently favor the annotations of the primary annotator (Annotator 1). Instead, it showed flexibility, occasionally agreeing with different annotators. This observation implies that the network learned a balanced representation of the data, effectively navigating divergent annotator styles and mitigating the impact of intra-annotator variability.

When applying the segmentation methods for WSI scoring, we observed only limited performance differences between the methods. This is likely because only a small proportion of the WSIs are near the decision boundary between different HER2 scores, meaning that variations in segmentation quality impact only a few cases. In contrast, the performance differences are more pronounced for the segmentation results, as the ROIs were specifically selected to capture regions with diverse characteristics.

Regarding the general behavior of the whole-slide HER2 scoring, our results reflect expected patterns in pathologist behavior, particularly regarding the HER2 1+ and 2+ classes. By reducing the scoring threshold for HER2 2+, we were able to mimic the scoring behavior of pathologist more closely, suggesting that pathologists may be inclined to label borderline HER2 1+ cases as 2+ to minimize the risk of missing patients who might benefit from targeted therapy. Nonetheless, confusion between these two classes persisted, likely due to persistent ambiguity in the staining intensities that separate HER2 1+ from 2+.

Our model demonstrated strong performance in discriminating HER2-positive from HER2-negative cases, misclassifying only one FISH-confirmed HER2-positive case as HER2 1+. Importantly, the model predictions would require a significantly reduced number of FISH test, as only 106 cases are predicted as HER2 2+, compared to 166 annotated cases. This reduction in equivocal cases has practical implications for clinical workflows, as it can save valuable time and resources while maintaining diagnostic accuracy. The model's ability to accurately classify HER2-low cases (with an accuracy of 92.45%) further underscores its potential utility in clinical practice, particularly in identifying patients who may benefit from emerging therapies targeting HER2-low breast cancer.

In addition, the segmentation analysis revealed substantial discrepancies among annotators, which is likely mirrored in the scoring annotations as well. Although scoring was performed by a single annotator and later reviewed by another, strong inter-rater variability indicates that a consensus-based evaluation could yield different results. Together, these findings highlight the complexity inherent in human-driven annotations and underscore the need for refined evaluation strategies, including consensus-driven benchmarks, to more accurately assess the performance and reliability of automated HER2 analysis.

## Conclusion

This work introduced a fully automatic HER2 tissue segmentation that can generate HER2 scores directly from WSIs without human intervention. By incorporating the exclusion of non-invasive tumor tissue in accordance with clinical guidelines, our approach provides interpretable and transparent results. Pixel-level predictions allow pathologists and researchers to visualize and validate how final scores are derived, offering clear insights into the tissue characteristics that shape diagnostic decisions.

Beyond its utility for HER2 scoring, our segmentation framework supports a wealth of future research opportunities. The ability to precisely delineate HER2 expression at a cellular and subregional level enables the exploration of tumor growth patterns and spatial heterogeneity. Such fine-grained analyses hold the potential to uncover new predictive markers for patient outcomes and treatment responses. As interest grows in personalized medicine, this line of inquiry may yield critical information that helps tailor therapies to individual patients' tumor profiles, ultimately improving care and optimizing treatment strategies.

## Declaration of generative AI and AI-assisted technologies in the writing process

During the preparation of this work, the authors used ChatGPT in order to improve the readability and grammar of the article. After using this tool, the authors reviewed and edited the content as needed and take full responsibility for the content of the published article.

## Declaration of competing interest

The authors declare that they have no known competing financial interests or personal relationships that could have appeared to influence the work reported in this article.
